# Orbital Apex Syndrome: a rare complication of herpes zoster ophthalmicus in a Ghanaian woman living with HIV

**DOI:** 10.4314/gmj.v55i4.14

**Published:** 2021-12

**Authors:** Esinam Ayisi-Boateng, Nana K Ayisi-Boateng, Kwadwo Amoah, Boateng Wiafe

**Affiliations:** 1 Eye Clinic, Kumasi South Hospital, Kumasi, Ghana; 2 Department of Medicine, Kwame Nkrumah University of Science and Technology, Kumasi, Ghana; 3 Infectious Disease Unit, University Hospital, Kwame Nkrumah University of Science and Technology, Kumasi, Ghana; 4 Operation Eyesight Universal, Accra, Ghana; 5 Watborg Eye Services, Kasoa, Ghana

**Keywords:** Acyclovir, Herpes Zoster Ophthalmicus, Human Immunodeficiency Virus, Orbital Apex Syndrome, Prednisolone

## Abstract

Herpes Zoster Ophthalmicus (HZO) usually affects the immunocompromised and aged. It results from the reactivation of latent varicella zoster infection in the trigeminal ganglia. Orbital apex syndrome (OAS) is a rare sequela of the disease and tends to be disfiguring and vision-threatening if not addressed. We report on a 43-year-old Ghanaian female living with Human Immunodeficiency Virus infection and on highly active antiretroviral therapy who presented with a 2-month history of a healed vesicular rash left side of the forehead and a droopy left upper eyelid. On examination, she had complete ptosis, visual acuity in the left eye was 6/36, and restricted mobility in all directions of gaze. On anterior segment examination using a slit lamp biomicroscope, the left eye had mild cornea oedema with keratic precipitates and relative afferent pupillary defect (RAPD). Intraocular pressure and posterior segment of the right eye were normal. Computed tomography (CT) Scan of the head was taken to rule out other causes of OAS. Patient was treated with oral acyclovir 400mg five times daily for 30 days, topical steroids and oral prednisolone 60mg daily for 30 days which was tapered. Ptosis improved significantly with mild supraduction and infraduction deficit. Visual acuity improved to 6/12 and all keratic precipitates cleared. The patient, however, developed a corneal scar from a possible neurotrophic ulcer after defaulting treatment for 11 months. OAS , as a rare sequalae of HZO, responds well to oral acyclovir and steroids. Prompt diagnosis and appropriate treatment, even at late presentation, yield positive outcomes.

**Funding:**

None declared

## Introduction

Herpes Zoster Ophthalmicus (HZO) occurs as a result of reactivation of latent herpes zoster virus which resides in the dorsal root ganglion of the ophthalmic division of the trigeminal nerve.^1^ The ophthalmic division further divides into the frontal, nasociliary and lacrimal branches with the frontal branch being the most affected.^2^ Reactivation of the virus usually occurs when the immunity is compromised either from disease or advancing age. 1,2 Ocular manifestations of the disease occur in 20%–70% of patients and ranges from blepharoconjunctivitis, keratouveitis, scleritis, endothelitis and lid complications such as entropion, ectropion and resultant exposure keratopathy.^1^,^3^ Corneal inflammation and opacification (49–89%) and anterior uveitis (43–92%) are the most common complications. Neurotropic keratopathy can also occur due to the reduced cornea sensitivity and this can eventually result in corneal scarring from unnoticed or poorly treated ulcers.^4^,^5^ Uncommon posterior segment complications of HZO include retinal perivasculitis, ischemic optic neuritis, and forms of necrotizing retinopathy.^1^ Rarely, it can result in an orbital apex syndrome (OAS). 6

Orbital apex syndrome (OAS) is a dysfunction of cranial nerve II, III, IV, V, and VI resulting in complete internal and external ophthalmoplegia.

The condition can be attributable to an inflammatory, infectious, neoplastic, vascular or traumatic cause.^7^ Orbital Apex Syndrome is a rare complication of herpes zoster ophthalmicus.^6^ Information on the condition in the published literature is also scanty. Verhaege et al in 2016 profiled 14 published case reports from 1966 to August 2015^8^ further reaffirming the rarity of the condition. By this report, we seek to add to the available knowledge on the condition.

## Case Report

**Figure 1:**
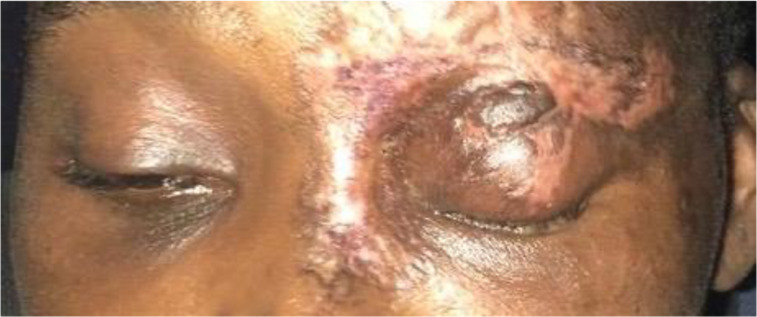
An image of the patient at presentation

A 43-year old Ghanaian woman diagnosed of HIV for 2 months and on lamivudine, efavirenz and tenofovir combination presented at the eye clinic with a 2-month history of a vesicular rash on the left side of the forehead which had healed completely (Figure 1). She also presented with reduced vision in the left eye and complete ptosis of the left upper eyelid. On examination, vision was 6/36 in the affected left eye, she had complete ptosis of the left upper eyelid and completely restricted ocular motility in all gazes.

Cornea sensation and sensation on the left side of forehead were reduced. Anterior segment examination of the left eye showed few pigmented keratic precipitates, mild cornea edema and relative afferent pupillary defect (RAPD). Intraocular pressure was 16 in both eyes and she had grade 1 nuclear sclerosis in the left eye. The optic nerve and macula appeared normal. Visual acuity in the right eye was 6/6 and all other examination findings of the right eye were essentially normal. A clinical diagnosis of Herpes Zoster Ophthalmicus was made pending investigations to identify the underlying cause. Patient could not afford a Magnetic Resonance Imaging (MRI) scan. Hence, a Computed Tomography (CT) scan of the head including the orbit was done, which was normal and ruled out other causes of orbital apex syndrome. She was given topical prednisolone 1% applied every 6 hours, oral acyclovir 400mg 5 times daily and oral prednisolone 60 mg daily for two weeks. Upon review, two weeks after initiation of therapy, the uveitis improved and all the keratic precipitates cleared.

**Figure 2:**
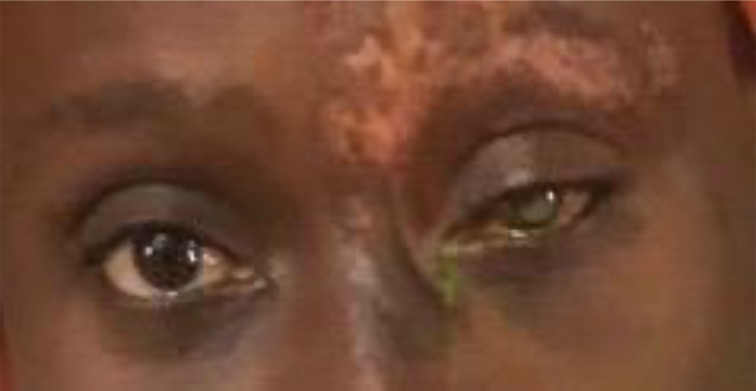
Image of the patient 12 months after treatment initiation

The visual acuity improved to 6/12 in that eye. However, the ptosis and ophthalmoplegia persisted. The same dosages of oral acyclovir and oral steroid were repeated for two weeks and the steroid was later tapered. The patient defaulted treatment and presented after 11 months with the ophthalmoplegia markedly resolved with mild ptosis and mild infra- and supraduction deficits. Visual acuity had reduced to 3/60 due to a corneal scar she developed (Figure 2). Ocular lubricants and topical steroids were administered when she presented with the corneal scar.

## Discussion

Until the advent of Human Immunodeficiency Virus (HIV), Herpes Zoster Ophthalmicus (HZO) was prevalent among the elderly. Increasing age leads to reduced specific cell mediated immunity to varicella zoster.^9^,^10^ Most cases of OAS following HZO occur among the elderly with the mean age of 68.1 years (range, 29–81 years) and the documented cases so far are predominantly women [73.3%].^11^,^8^ The reason for this female preponderance is worth exploring. Our patient being a 43-year old female, falls below the mean age. HZO has previously been reported in a much younger 29-year old HIV positive female.^6^ The occurrence of HZO in a young patient therefore warrants a test for HIV.^12^ As compared to the general population, HIV-infected individuals have 15–25 times greater prevalence of herpes zoster infection and 6.6 times risk of HZO.^13^,^14^

HIV infection may be associated with a wide spectrum of ocular manifestations and in a number of asymptomatic individuals, these manifestations may be an early sign of infection.^15^ Some of these ocular signs tend to occur in HIV patients with low CD4 counts. A study in Ethiopia reported a high incidence of HZO in HIV infected patients with CD4 count between 200 – 499 cells/µl.^15^ An HIV-infected individual may experience ocular inflammation subsequent to HAART initiation as a result of Immune Reconstitution Inflammatory Syndrome (IRIS).^16^ However the incidence of ocular complications of HIV infection has decreased particularly in the developed countries because of the use of HAART.^6^

In the pre-HAART era, the prevalence of HIV-related ocular manifestations was higher than post-HAART era. However, the Ethiopian study showed that ocular manifestations were more common in patients on HAART than in those not on HAART.^15^ They attributed this to the low CD4 count of patients who were started on HAART. Our patient's CD4 count results were not available. This may be attributable to frequent breakdown of laboratory equipment, shortage of reagents and difficulty in accessing other testing sites in low resource settings. 17 Apart from patients' clinical conditions serving as an estimate of advanced HIV infection or low CD4 count, total lymphocyte count, proteinuria and leukocytouria may be useful substitutes for CD4 count.^18^,^19^

HZO has a wide spectrum of presentation and by our experience with this case, it is important to highlight that affected patients may exhibit only some aspects of the disease. Patients with HZO with concomitant HIV have severe and prolonged ocular complications 20 and late presentation can be associated with poor outcome.^10^ The mean interval between rash and ophthalmoplegia is 10 days.^8^ Our patient reported to the eye clinic two months after herpes zoster rash on forehead had virtually healed and she had developed anterior uveitis as well as orbital apex syndrome. Nonetheless, she had been on antiretrovirals (ARVs) for the same duration of symptoms. Initiation of antiviral therapy < 72hrs after onset of rash reduces the risk of progression to late stage ocular complications.^21^,^1^

The diagnosis of OAS is largely based on a detailed history and thorough clinical examination. The most appropriate radiological investigation is magnetic resonance imaging (MRI)^7^ but this is not readily available and affordable to patients in resource-limited settings like Ghana. Typical MRI findings are extraocular muscle thickening and enhancement of optic nerve sheath and orbital apex.^8^ These changes may persist even after clinical signs have resolved.^7^ Computed tomography (CT) scan taken for our patient was normal similar to what was found in a Chinese patient^22^ but contrary to proptosis and generalized swelling of the extraocular muscles detected in a 63-year old woman with a similar presentation.^23^

The definitive treatment of HZO-related OAS remains unclear. However, acyclovir and steroids, be it oral or intravenous, have both shown effectiveness with a more positive tilt towards intravenous therapy.^24^ This formed the basis of treatment given to our patient. The topical steroid was administered on account of the anterior uveitis the patient presented with. Partial or total resolution of ophthalmoplegia usually takes 2 weeks to 36 months and has been reported in 76.5% of cases.^25^ In our patient, this took approximately 4 weeks, despite her late presentation. Her visual acuity improved initially but later worsened due to a corneal scar she developed from a possible or likely neurotrophic ulcer whose healing was hampered after she defaulted treatment. Neurotrophic ulcer of the cornea with extensive scarring and neovascularization have been reported elsewhere.^26^ This case report, though limited by a single patient experience, underscores the fact that under the cover of HAART, oral acyclovir plus oral steroids have a favorable prognosis in OAS following HZO in an HIV patient. The use of steroids for the treatment of OAS in immunocompromised patients remains a subject of debate.^6^ This can be explored in a bigger study among a cohort of patients with OAS. However, this can be challenging since OAS is rare.

## Conclusion

OAS is gradually becoming a recognized complication of HZO and ophthalmologists and eye care professionals should have a high index of suspicion to enable early detection and institution of therapy. Our case report underscores the fact that therapy can be initiated for OAS regardless of the stage of presentation. However, such patients should be counseled on treatment adherence and must be closely followed up since they are prone to HZOrelated reduced corneal sensitivity which can threaten their vision.
